# The role of exercise in aromatase inhibitor‐induced arthralgia

**DOI:** 10.1002/pmrj.13193

**Published:** 2024-05-23

**Authors:** Kerstin Yu, Pauline Portes, G. Stephen Morris, Laura Huang, Elizabeth R. Felix, Gary J. Farkas, Diana Molinares, Eduard Tiozzo

**Affiliations:** ^1^ University of Miami Miller School of Medicine Miami Florida USA; ^2^ Department of Physical Therapy Wingate University Wingate North Carolina USA; ^3^ Department of Physical Medicine & Rehabilitation University of Miami Miller School of Medicine Miami Florida USA; ^4^ Mount Vernon Rehabilitation Medicine Associates Alexandria Virginia USA

## Abstract

Aromatase inhibitors are prescribed in breast cancer due to their associated lower rate of cancer recurrence compared to tamoxifen. However, aromatase inhibitor‐induced arthralgia (AIIA) is one of the leading causes of treatment nonadherence, increasing the risk of cancer recurrence. The pathophysiology of AIIA is poorly understood, and although current recommendations for AIIA include lifestyle changes and analgesics depending on the severity of symptoms, there is no established effective treatment. The aim of this study is to explore the presentation and mechanism of AIIA and investigate the feasibility and efficacy of different exercise interventions (aerobic, resistance, aerobic and resistance combined, and yoga or tai chi) in patients with AIIA to guide the development of formal exercise prescription guidelines. Findings indicate that a mixed‐modality regimen of aerobic and resistance exercises is feasible and safe and may serve the most benefit in improving joint pain, functionality, and quality of life. More specifically, the weekly regimen should consist of 150 min of aerobic exercise with two sessions of at least six resistance exercises, 8 to 12 repetitions, three sets each. Supplementary yoga and tai chi may be recommended twice a week depending on a patient's target symptoms. Yoga was associated with improved physical functionality, whereas tai chi was related to improvements in mental health. However, the feasibility and impact of combined aerobic and resistance exercise protocols with yoga or tai chi in our target population were not investigated in this review. The use of large, randomized controlled trials is recommended for future studies.

## INTRODUCTION

Aromatase inhibitors (AIs) are the treatment of choice for patients with estrogen‐receptor positive breast cancer. These drugs inhibit the aromatization necessary for estrogen formation, effectively decreasing estrogen levels. They are prescribed for both active disease and remission, often requiring 5 to 10 years of treatment.^1^ Tamoxifen, a nonsteroidal selective estrogen‐receptor modulator, has been largely replaced by third‐generation AIs such as letrozole, exemestane, and anastrozole due to the greater association of disease‐free survival with AIs than tamoxifen. More specifically, breast cancer recurrence rates are reduced by a third with AIs compared to tamoxifen and by half compared to no treatment.^2^, ^3^

Unfortunately, AI therapy is associated with adverse events and a high rate of discontinuation in the first few years. One complication is a phenomenon known as aromatase inhibitor‐induced arthralgia (AIIA), which occurs in 20%–74% of patients.^4^, ^5^ AIIA symptoms include morning stiffness and joint pain affecting the hands, wrists, knees, lower back, hips, shoulders, and feet, with the fingers being most commonly affected.^6^ Among women with early‐stage breast cancer receiving AI therapy, Henry et al. reported that nearly a quarter of patients discontinued original AI therapy due to AIIA and did so at a median time of 6 months after treatment initiation.^7^ Among patients who switched to a different AI due to AIIA, only 38% were able to tolerate the second AI and only did so for a median of 13 months. These high rates of AI discontinuation attenuate the efficacy of breast cancer treatment, increase the risk of cancer recurrence, and contribute to greater mortality rates.^8^, ^9^

Given the critical role AIs play in disease management, it is clinically important to maximize the use of this treatment. The current recommendations for AIIA management include lifestyle changes such as physical exercise and dietary modifications, and the use of nonsteroidal anti‐inflammatory drugs or other analgesics depending on the severity of symptoms.^10^

As exercise is a recommendation in AIIA management, clinician understanding of exercise prescription is important. Therefore, the purpose of this review is to investigate the presentation and pathophysiology of AIIA and explore the feasibility and efficacy of different exercise modalities in patients with breast cancer experiencing AIIA to ultimately guide the development of formal exercise prescription guidelines for this condition.

## PRESENTATION AND PATHOPHYSIOLOGY OF AIIA

The characteristic presentation of AIIA is joint pain, stiffness, and achiness commonly affecting the knees, hands, wrists, and shoulders.^11^ To be classified as AIIA, the arthralgia must develop or worsen during AI therapy, improve or resolve within 2 weeks of AI discontinuation, and return upon resuming the AI.^5^ The joint pain may be accompanied with stiffness or muscle weakness and can impair a patient's ability to perform activities of daily living and work‐related tasks.^11^, ^12^

Although the pathophysiology of AIIA is not completely understood, a few main ideas recur in the literature. For example, AI therapy results in estrogen deprivation, which has been implicated in the mechanism of AIIA. Evidence for this mechanism originates from the notion that estrogen deprivation is responsible for the arthralgia experienced during menopause.^13^ Arthralgia is also seen in women treated with gonadotropin‐releasing hormone analogs, which suppress estrogen levels.^14^, ^15^ This link between estrogen deprivation and arthralgia can be explained by estrogen's physiological effects, which are shown to have anti‐inflammatory properties, modulate nociception, and even suppress cartilage degradation.^16^, ^17^, ^18^ Estrogen is known to suppress the production of proinflammatory cytokines such as tumor necrosis factor‐α and interleukin‐1, which has been associated with being a crucial mediator in inflammatory cartilage and bone degradation in people with rheumatoid arthritis.^19^ Thus, a reduction in estrogen via aromatase inhibition may increase pain, inflammation, and cartilage damage.

Local inflammation in the joint may also be implicated in the mechanism of AIIA. In fact, aromatase is expressed in chondrocytes and synovial cells and has been shown to reduce proinflammatory interleukin‐6 expression in the joint space.^12^ This concept is largely supported by radiographic studies displaying tenosynovial changes consistent with inflammation in patients taking AI.^20^, ^21^ Additionally, sonographic evaluations of patients experiencing AIIA showed higher rates of joint effusions than the general population.^20^

The role of systemic inflammatory cytokines in the mechanism of AIIA has also been explored, with some studies showing an increased level of circulating inflammatory markers in patients with AIIA.^22^, ^23^ A cross‐sectional study of 203 patients with breast cancer receiving AI therapy reported an association between the presence of moderate‐to‐severe arthralgia and increased levels of inflammatory biomarkers, suggesting a possible underlying systemic inflammation.^23^ Two other studies, however, showed no change in systematic inflammatory markers before and after treatment with AI in women experiencing AIIA.^24^, ^25^

Overall, the pathophysiology of AIIA appears largely multifactorial. The proposed mechanisms that recur in the literature include estrogen deprivation and local and/or systemic inflammation.

## CURRENT RECOMMENDATIONS FOR MANAGEMENT OF AIIA

Counseling and education in the early stages of AIIA management are important due to the possibility of AIIA causing poor medication adherence, which can lead to an increased risk of cancer recurrence. Younus and Kligman recommend that follow‐up visits be scheduled at 2 and 6 months from the date of AI initiation to discuss patient symptoms, as associated symptoms usually appear approximately 2 months from treatment initiation and peak around 6 months.^8^ Current clinical recommendations for management of AIIA include lifestyle modifications such as regular exercise and weight loss, dietary modifications such as reduction of alcohol consumption and increased intake of calcium and vitamin D, and the addition of analgesics such as acetaminophen, nonsteroidal anti‐inflammatory drugs, or even opioids depending on the severity of symptoms.^26^

The American College of Sports Medicine (ACSM) endorses a combined aerobic and resistance regimen for individuals who are being treated for cancer. The specific exercise recommendations including minutes per session, days per week, sets and repetitions vary depending on the desired goal (Table 1).^27^ Although management strategies for AIIA list exercise as an option, specifications for modality, intensity, duration, and frequency of exercise remain undefined.^8^

**TABLE 1:** Goal‐Directed American College of Sports Medicine Guidelines for Exercise and Cancer.

Therapy goal	Dose of combination (aerobic + resistance)
Cancer‐related fatigue	30 min of aerobic exercise 3x/week at moderate intensity + resistance training 2x/week at moderate intensity; 2 sets of 12–15 repetitions for major muscle groups
Health‐related quality of life	20–30 min of aerobic exercise 2–3x/week at moderate intensity + resistance training 2x/week at moderate to vigorous intensity; 2 sets of 8–15 repetitions for major muscle groups
Physical function	20–40 min of aerobic exercise 3x/week at moderate to vigorous intensity + resistance training 2–3x/week at moderate to vigorous intensity, 2 sets of 8–12 repetitions for major muscle groups
Anxiety and depression	20–40 min of aerobic exercise 2–3x/week at moderate to vigorous intensity + resistance training 2x/week at moderate to vigorous intensity, 2 sets of 8–12 repetitions for major muscle groups

It is estimated that only 35% of breast cancer survivors meet the general guidelines of 150 minutes of weekly exercise recommended by the Centers for Disease Control and Prevention.^28^ Meeting the minimum amount of prescribed exercise remains a challenge even for cancer‐free individuals. The percentage of adults in the United States who meet the national guidelines of 150 minutes of moderate intensity per week is 53%, and the percentage of the same adults who meet the national guidelines for aerobic and muscle‐strengthening activity is only 23%.^29^, ^30^, ^31^ Therefore, feasibility and adherence should be considerations in research and development of exercise guidelines for AIIA.^32^

## SEARCH STRATEGY

A search of the PubMed electronic database was executed. Search terms related to the topics of interest were used in different combinations, including “aromatase inhibitors” or “aromatase inhibitor”, and “breast cancer” or “breast carcinoma” or “breast neoplasm”, and “exercise” or “aerobic” or “resistance” or “yoga” or “tai chi” or “walking” or “running”, as well as “arthralgia” or “joint pain”.

## INCLUSION AND EXCLUSION CRITERIA

To be included, articles had to involve the population of interest: patients with breast cancer and AI‐associated joint pain. The article had to include one of the exercise interventions of interest and measurements of its effects on the symptoms of AIIA, physical functionality, or quality of life (QoL). Chosen articles were all in English. Case reports and case series were excluded.

## AEROBIC EXERCISE

Aerobic exercise is shown to improve inflammation, pain, anxiety, and depression, which are symptoms commonly associated with breast cancer and AI therapy.^33^ A one‐time strenuous bout of exercise increases oxidative metabolism and produces a pro‐oxidant environment in individuals who are not conditioned, but routine physical activity can actually alleviate an inflammatory state.^34^, ^35^ Positive mental health benefits such as the reduction of anxiety, depression, and stress can additionally be achieved with aerobic exercise.^36^ Studies have reported that regular aerobic exercise is associated with lower sympathetic nervous system and hypothalamic–pituitary–adrenal axis reactivity, which plays an important role in developing adaptive responses to physical and psychological stressors.^37^, ^38^, ^39^, ^40^ Depression and anxiety have been linked to dysregulations in the hypothalamic–pituitary–adrenal axis, and aerobic activity can mitigate such dysregulation.^41^, ^42^ When discussing the benefits of aerobic exercise in patients with AIIA, the feasibility and efficacy of aerobic interventions need to be examined and discussed to develop more specific exercise guidelines for this population.^27^

The feasibility of a 6‐week walking program was examined in elderly survivors of breast cancer experiencing AIIA.^43^ The self‐directed program, called the Arthritis Foundation's Walk With Ease, was originally developed for people with joint pain and stiffness. In people with arthritis, the walking program has been shown to reduce arthritis symptoms and increase physical performance such as balance, strength and walking speed.^44^, ^45^ In this study, all participants (*n* = 20) were 65 years and older, with a diagnosis of breast cancer (stage I–III), had self‐reported joint pain/stiffness, and were on AI therapy for at least 3 months. The sample was primarily White (85%) with a mean age of 71 years and a mean body mass index of 29 kg/m^2^. Half of the participants were able to meet and tolerate 150 minutes of aerobic activity a week, as recommended by the national guidelines for the population regardless of breast cancer status. Although not statistically significant, mean joint pain improved by 10%, fatigue by 19%, and joint stiffness by 32% after the 6‐week walking intervention. These findings suggest that the walking program is feasible for older women with breast cancer experiencing AIIA. Future studies should use a larger sample size and explore methods of increasing adherence to walking interventions.

A randomized‐controlled study examined the feasibility of a Nordic walking program in women experiencing AIIA.^46^ Nordic walking is an aerobic exercise consisting of walking with single or double arm poling movements, and it has been associated with a reduced joint load and an increased aerobic endurance and muscle strength capacity.^47^, ^48^, ^49^ The study sample (*n* = 40) was exclusively White, and the mean age was 63 years with an average of 36 months from diagnosis, 27 months on AI therapy, and 22 months experiencing arthralgia. Participants were randomized to the intervention (enhanced usual care plus walking program) or control (enhanced usual care only). Enhanced usual care included standard care plus biweekly calls to check for any new injury, pain, or lymphedema. Participants in enhanced usual care also received support and encouragement through these biweekly phone calls as well as an informational booklet on physical activity and health benefits. They were additionally requested to complete an exercise diary. The walking program was a total of 12 weeks: 6 weeks of supervised group Nordic walking training once a week, followed by 6 weeks of independent walking sessions. Intervention participants were able to sustain increased activity levels over a 12‐week period with no increases in lymphedema or injuries, demonstrating the feasibility and safety of Nordic walking in our target population. This study was not adequately powered, but positive trends were observed in pain, depression, self‐efficacy for managing pain, and QoL in both intervention and control groups. Possible explanations for the clinical improvements in both groups include increased physical activity among all participants, the observer bias, or the support and encouragement provided to both groups. Adequately powered, larger, randomized controlled trials (RCTs) are recommended to determine whether there is a difference between the addition of a Nordic walking program to enhanced usual care for patients with AIIA.

A phase II trial investigated the impact of a 6‐week, self‐directed walking program on AIIA in women with breast cancer.^50^ Participants (*n* = 62) were postmenopausal with a mean age of 64 years and body mass index of 30 kg/m^2^ or higher and were randomized to a walking intervention of at least 150 minutes a week or wait list control. Self‐reported measures included Western Ontario and McMaster Universities Osteoarthritis Index (WOMAC) subscales for pain, stiffness, and function/difficulties with activities of daily living. Control and helplessness over disease‐related outcomes were assessed using the Rheumatology Attitudes Index. At the end of the 6‐week walking program compared to baseline, the intervention group demonstrated statistically significant improvements in mean walking minutes per week from 32.49 to108.7 (+234.6%), stiffness from 4.43 to 3.49 (−21.2%), function/difficulties with activities of daily living from 24.39 to 17.69 (−27.4%), WOMAC Total from 35.99 to 27.24 (−24.3%), and perceived helplessness score from 2.34 to 1.92 (−18.4%). At 6 months post‐intervention, mean walking minutes per week had decreased from post‐intervention by 34.99 (−34.60%), and the perceived helplessness score returned to near baseline values, but the improvements in stiffness, function, and WOMAC Total were maintained.

In summary, walking as aerobic exercise can be feasible and safe in women with breast cancer and experiencing AIIA. Aerobic exercise seems to produce more favorable outcomes in pain, functionality, and QoL. Larger and longer aerobic interventions in the form of RCTs as well as methods to increase intervention adherence should be explored to determine whether clinically significant improvements can be achieved in the same patient population.

## RESISTANCE EXERCISE

Resistance exercise has been associated with increased muscle mass and strength and maintenance of bone density.^51^ Besides physical health associations, resistance training is also beneficial for mental health.^52^ To our knowledge, there are no published studies investigating only resistance exercise interventions in individuals with breast cancer and AIIA. Given the support for combined resistance and aerobic exercise in AIIA, the effects of resistance exercise alone in AIIA are worth evaluating.

## AEROBIC AND RESISTANCE EXERCISE IN COMBINATION

The ACSM endorses a combined exercise program of aerobic and resistance training for improving cancer‐related fatigue, health‐related QoL, physical function, anxiety, and depression in patients with cancer. There are studies that have explored the association between combined exercise and arthralgia in survivors of breast cancer receiving AI therapy.

A longitudinal pilot study (*n* = 26) examined the impacts of a combined aerobic and resistance program on arthralgia and functional performance in patients who are postmenopausal with breast cancer treated with AI.^53^ The 8‐week, home‐based combined program additionally taught participants how to warm up and cool down. The aerobic exercises included participant‐selected cardiovascular sessions with a goal of achieving and maintaining 55%–65% of maximum heart rate. The resistance exercises included upper and lower body using exercise bands with video instructions. Because hand pain is a major concern in those with AIIA, study participants were also instructed on how to perform different hand exercises. The mean age of participants was 63 years, and the mean time since cancer diagnosis and treatment was 18 months. The combined program demonstrated statistically significant improvements in several indices of QoL, exemplified by the improvements in arthritic pain, physical activity, arm function, and dexterity. Participants reported decreased pain in all joints measured, with the largest absolute changes observed in the shoulders, fingers/thumb, and lower back. Functional performance in grip strength and biceps curls were significantly improved by 14% and 51%, respectively. Signs of depression expressed as the percentage of participants at risk decreased from 19% at the beginning of the study to 3% at the end of the study, indicating an 84% improvement. No exercise‐related adverse events were reported. It is also important to note that the study was unable to determine whether the observed improvements in depression symptoms and QoL were direct results of the exercise program, increased social interaction through the intervention, or secondary to the significant reduction in joint pain.

A large, randomized‐controlled exercise trial called HOPE (Hormones and Physical Exercise) examined the effects of a 12‐month combined exercise intervention on AIIA in 121 survivors of breast cancer.^54^ Participants were randomly assigned to a combined exercise program or attention control receiving health education for 12 months. The mean age of participants was 61 years with 88% being White and 60% diagnosed with stage I breast cancer, with an average time between diagnosis and enrollment of 3 years. The exercise intervention included 150 minutes per week of aerobic exercise (brisk walking on a treadmill with the option of using a stationary bicycle) and twice‐a‐week supervised strength training (bench press, latissimus pull‐down, seated row, leg press, leg extension, and leg curl) performed for 8 to 12 repetitions for three sets each exercise. The weight was increased by the smallest possible increment if a participant was able to lift the same weight 12 times during each set for two sessions. Arthralgia was assessed using the Brief Pain Index, which uses an 11‐point scale measuring self‐reported pain intensity. Lower‐extremity joint symptoms (pain, stiffness, and physical function) in the past 7 days were assessed using the WOMAC index, and upper‐extremity joint symptoms using the Disabilities of the Arm, Shoulder and Hand questionnaire. In all the scales, lower scores indicate improvements and higher scores worsening of the symptoms. At 12 months, mean worst joint pain scores demonstrated a statistically significant decrease of 1.6 points (−29%) in the exercise group and increase of 0.2‐point (+3%) in the usual care group (difference, 1.8). Lower‐ and upper‐body symptoms exhibited statistically significant improvements as well. At 12 months, the score for lower extremities decreased by 9.4 points (−37%) in the exercise group and increased by 0.5 point (+2%) in the control group (difference, 9.9). Also at 12 months, the upper‐extremity score significantly decreased by 6.7 points (−33%) in the intervention group and increased by 1.3 points (+12%) in the usual care group (difference, 8.0). The effects of exercise on arthralgia were found to be non‐dose dependent. This indicates that 150 minutes of aerobic exercise and two resistance sessions per week may be effective for improving AI‐associated arthralgia, if performed over 1 year. The resistance sessions should include at least six exercises performed for 8 to 12 repetitions for three sets each, with gradual increases in weight over time, as indicated in this trial.

A HOPE ancillary study evaluated the role of a 12‐month exercise intervention program on breast cancer‐related, endocrine‐related, and fatigue‐related QoL among the same participants.^55^ At 12 months, the exercise group demonstrated statistically significant improvements in breast cancer‐specific, endocrine‐specific, and fatigue‐specific QoL measures compared to the usual care group.

In summary, combined aerobic and resistance exercises can be feasible and safe in AIIA management in patients with breast cancer. In addition, this combined exercise regimen has been shown to be effective in improving joint pain, depression, and QoL among the same patient population. Current literature suggests that combined exercise training can be more effective than aerobic exercise alone in reducing AI‐associated symptoms, thus potentially improving medication adherence and risk of cancer recurrence in women with breast cancer.

## YOGA AND TAI CHI

Alternative exercise modalities such as yoga and tai chi have gained widespread popularity and increasing interest in their possible health benefits. Yoga has been shown to improve mental health while diminishing feelings of depression, anxiety, and pain‐associated disability.^56^, ^57^ Studies have also associated yoga with improvements in low back pain, knee osteoarthritis, and carpal tunnel syndrome in noncancer populations.^58^, ^59^, ^60^, ^61^ Zou et al. reported in a systematic review and meta‐analysis of RCTs that stress reduction and subsequently improved QoL may be attributable to a down‐regulation of sympathetic‐vagal balance modulated by mind–body exercises such as yoga and tai chi.^62^ With the emerging benefits of yoga and tai chi in the non‐cancer population, the impacts of these two modes of exercise can reasonably be expected to extend to survivors of breast cancer.

In a recent systematic review of RCTs, Danhauer et al. reported that yoga improved overall QoL and physical fatigue both during and after cancer treatment that included surgery, chemotherapy, radiation therapy, or hormonal therapy.^63^ In another study, survivors of breast cancer on AI (*n* = 95) or tamoxifen (*n* = 72) were randomized to 4 weeks of yoga or standard care. General pain, muscle aches, and total physical discomfort from pre‐ to post‐intervention demonstrated statistically significant improvements with yoga compared to standard care, with 90% of participants reporting reduced severity in all three domains.^64^ A small RCT in women who completed breast cancer treatment (*n* = 21) showed significant improvements in functional capacity and QoL after 12 weeks of tai chi training, compared to standard care.^65^ All participants had completed surgical treatment and a majority received at least another treatment modality: chemotherapy, radiation therapy, or hormonal therapy.

The feasibility and efficacy of yoga on AI‐associated joint pain and QoL were explored in a small study.^66^ The participants (*n* = 10) were postmenopausal survivors of breast cancer who had completed chemotherapy and/or radiotherapy and reported AI‐associated joint pain. Iyengar yoga classes were held for 90 minutes twice weekly for 12 weeks specifically for study participants. This specific type of yoga emphasizes proper alignment and precise technique by placing muscles under prolonged tension and is associated with improved strength and flexibility over time. The mean number of the 24 classes attended was 13.8 (58%) with a range of 6 to 20. Statistically significant improvements were observed in overall pain severity and interference, fatigue severity, stiffness of the hand, and pain, stiffness, and impaired function of the knee/hip. Improvements in hand pain and function, depressive symptoms, fatigue interference, and insomnia were not significant. Finally, 70% of the participants rated the yoga classes “very” or “extremely” helpful in improving pain and 100% would recommend the classes to other women with AI‐associated pain. With an array of pain and functional improvements and an average attendance of 58% over the course of 12 weeks, yoga should be considered feasible and beneficial in the management of AIIA.

A separate study (*n* = 10) examined the impact of yoga on objective functional outcomes, pain, and health‐related QoL in postmenopausal survivors of breast cancer currently receiving AI therapy and experiencing AIIA.^67^ The intervention was an Iyengar yoga program, offered twice a week for 8 weeks. Overall adherence to the program was 80%, and no development or worsening of lymphedema or other adverse events were reported, proving Iyengar yoga to be feasible and safe. From pre‐ to post‐intervention, statistically significant improvements were observed in balance (54%), flexibility (31%), overall functional limitations (58%), joint pain severity (28%), and health‐related QoL (18%).

Tai chi has been historically linked to improvements in strength and flexibility as well as mental health. The impact of tai chi on function, pain and QoL in survivors of breast cancer with AIIA was examined in a feasibility study.^68^ Participants (*n* = 12) were postmenopausal women with a history of stage I–III breast cancer reporting AIIA. The intervention consisted of 1‐hour tai chi classes twice a week for 8 weeks. At the end of the intervention, adherence to classes was 75% with no documented adverse events, demonstrating tai chi to be feasible and safe. Participants experienced statistically significant improvements in anxiety, depression, emotional well‐being, fatigue, and the sit‐and‐reach. Although participants experienced improved mental health‐related QoL with the tai chi trial, the intervention did not appear to have a significant impact on joint pain or physical well‐being.

In summary, yoga and tai chi appear to be feasible and safe in postmenopausal women with a history of breast cancer receiving AI therapy and experiencing AIIA. In comparing yoga and tai chi, yoga appears to produce greater improvements in joint pain, functionality and health‐related QoL, and tai chi in depression and anxiety in study participants. Overall, larger RCTs are needed to determine the full scope of benefits yoga and tai chi can provide to survivors of breast cancer experiencing AIIA.

## DISCUSSION

All exercise modalities reviewed (Table 2) were shown to be feasible and safe for patients with breast cancer experiencing AIIA.

**TABLE 2:** Summarized findings of reviewed sources.

Intervention type	Study, origin, and sample size	Summary of intervention	Target population	Summary of findings
*Aerobic*	Nyrop et al.^50^ United States *n =* 20	Feasibility study involving a self‐directed, 6‐week walking program with minimum 30 min of walking 5 days a week (150 min per week)	Age > 65; Stage I–III breast cancer; ≥3 months of AI therapy; self‐reported joint pain/stiffness	Proportion walking 150 min/week increased from a baseline of 21% to 50% at 6 weeks (*p* < .001). Mean joint pain decreased 10% (*p* = .63), fatigue decreased 19% (*p* = .31), joint stiffness decreased 32% (*p* = .07) at 6 weeks.
	Fields et al.^46^ United Kingdom *n* = 40	RCT of walking intervention (6‐week supervised group Nordic walking training, followed by 6 weeks 4 × 30 min/week independent Nordic walking) in addition to enhanced usual care or enhanced usual care only	Women on AI therapy reporting joint symptoms and without metastatic disease, not already undertaking Nordic walking, and not unable to exercise due to mobility issues.	Adherence was >90% for weekly supervised group Nordic walking. Positive trends were observed in pain, depression, self‐efficacy for managing pain, and QoL in both intervention and control group.
	Nyrop et al.^50^ United States *n* = 62	RCT of 6‐week, home‐based, self‐directed walking program (150 min per week) or wait list control	Survivors of stage 0–III breast cancer, receiving AI for at least 4 weeks, ≥3 self‐reported joint pain on a 5‐point scale, stiffness, or achiness on a 5‐point scale (5 indicating “at its worst”), and exercising ≤150 min per week	Compared to the control group, the intervention group demonstrated significant improvements in increased walking minutes per week (*p* < .01), stiffness (*p* < .05), function/difficulty with activities of daily living (*p* < .01), and perceived helplessness score (*p* < .01). At the 6‐month follow‐up, only the improvements in stiffness and function were maintained.
*Aerobic and resistance*	DeNysschen et al.^53^ United States *n* = 26	Pre‐post pilot study involving an 8‐week, home‐based program consisting of resistive and aerobic exercises	Postmenopausal women with history of estrogen receptor positive breast cancer on at least 1 month of AI therapy with reports of mild to moderate arthralgias	Significant improvements in several indices of QoL, including arthritic pain (*p* ≤ .01), physical activity (*p* ≤ .01), arm function (*p* ≤ .02), and dexterity (*p* ≤ .04). Decreased pain in all joints was reported. Functional performance in grip strength and biceps curls was improved (*p* ≤ .01). Signs of depression indicated an 84% improvement.
	Irwin et al.^54^ United States *n* = 121	RCT of 12‐month combined exercise intervention (150 min per week of aerobic exercise and twice‐a‐week supervised strength training) or attention control receiving health education	Women with breast cancer receiving an AI for at least 6 months, reporting ≥3 of 10 for worst joint pain on the Brief Pain Index, and reporting <90 min per week of aerobic exercise and no strength training	At 12 months, scores for worst joint pain, pain severity and interference, upper‐extremity joint symptoms, and lower‐extremity joint symptoms decreased significantly post‐intervention in women randomly assigned to exercise compared to those in the control group (all *p* < .001). All scores were seen to be increased in the control group.
	Baglia et al.^55^ United States *n* = 121	*Ancillary study to above*	*Ancillary study to above*	Post intervention, the exercise group demonstrated greater improvements in the overall QOL measures and breast cancer‐specific (*p* = .02), endocrine‐specific (*p* < .001), and fatigue‐specific (*p* < .001) subscales compared with the usual care group.
*Yoga or tai chi*	Galantino et al.^68^ United States *n* = 10	Iyengar yoga program offered twice a week for 8 weeks	Postmenopausal women with stage I to III breast cancer on AI experiencing AIIA	Overall adherence to the program was 80%. Post intervention, significant improvements in balance (*p* = .048) flexibility (*p* = .009), overall functional limitations (*p* < .05), joint pain severity (*p* < .05), and health‐related QoL (*p* < .05).
	Galantino et al. 2013 United States *n* = 12	Group tai chi practiced for 1 h twice a week for 8 weeks	Postmenopausal women with a history of stage I–III breast cancer reporting AIIA	Adherence to classes was 75%. Participants experienced significant improvements in anxiety (*p* = .003), depression (*p* = .020), emotional well‐being (*p* = .027), fatigue (*p* = .030), and the sit‐and‐reach (*p* = .016) at the end of intervention. No significant impact on joint pain or physical well‐being.
	Jacobsen et al.^66^ United States *n* = 10	Iyengar yoga classes were held for 90 min twice weekly for 12 weeks	Postmenopausal female breast cancer survivors who had completed chemotherapy and/or radiotherapy (as applicable) and reported joint pain that started or worsened after AI initiation (≥4 on a 10‐point scale in the past week)	Mean number of the 24 classes attended was 13.8 (58%). Significant improvements in overall pain severity and interference, fatigue severity, stiffness of the hand, and pain, stiffness, and impaired function of the knee/hip (*p* < .05). Improvements in hand pain and function, depressive symptomatology, fatigue interference, and insomnia were not significant.

Abbreviations: AI, aromatase inhibitor; AIIA, aromatase inhibitor‐induced arthralgia; QoL, quality of life; RCT, randomized controlled trial.

Current evidence suggests that exercise protocols combining both aerobic and resistance exercises may be more effective in managing AIIA and overall QoL in patients with breast cancer than independent treatment with these modalities.^54^, ^55^ In review, the intervention of aerobic exercise alone in the form of a walking program in our target population appeared to improve AI‐associated pain and stiffness, although not all results were statistically significant.

With resistance training alone, significant improvements in muscle strength, joint range‐of‐motion, fatigue, pain, and QoL have been demonstrated in general survivors of breast cancer regardless of AIIA status.^69^, ^70^ However, there are no specific conclusions that can be drawn on resistance exercise alone on AIIA due to the lack of research with this exercise modality alone in our target population.

Combined programs of aerobic and resistance exercises were associated with significant improvements in joint pain, functionality, and QoL. Specifically, the HOPE study by Irwin et al. demonstrated significant improvements with 150 minutes of aerobic exercise combined with two resistance sessions of at least six exercises per week, 8 to 12 repetitions for three sets each, when performed for over a year.

Both yoga and tai chi have also proven feasible and safe, with the statistically significant improvements with yoga associated with increased physical functionality, while the positive effects of participating in tai chi appear to be more related to statistically significant improvements in the mental health in this patient population.^66^, ^67^, ^68^, ^69^ Based on existing studies, the intervention should be performed twice a week for at least 8 to 12 weeks. However, it is important to note that the combined effects of yoga and/or tai chi in addition to a regimen of aerobic and resistance exercises were not specifically explored in our target population for this study.

## CONCLUSION AND FUTURE DIRECTIONS

AIIA is one of the leading causes of cancer therapy discontinuation or poor adherence, indicating the importance of exploring exercise modalities that can improve AI‐associated joint pain and cancer prognosis. The adverse effect of joint pain experienced with AI continues to be variably managed without formal exercise guidelines for this patient population.

Given the available body of evidence, our recommendation to improve AIIA and AI adherence, thus reducing the risk of cancer recurrence in our target population, is a combination program such as the one employed in the HOPE study: a regimen of 150 minutes of aerobic exercise with two sessions of at least six resistance exercises (performing each for 8 to 12 repetitions for three sets) per week, performed for at least 12 months. Although the ACSM guidelines for exercise and cancer (Table 1) indicate only two sets of repetitions per resistance exercise twice a week, it is important to note that these guidelines are not specific to AIIA. The discrepancy between the number of sets per resistance exercise between the HOPE study and ACSM guidelines suggests that performing a large, randomized‐controlled combined exercise trial using two sets of repetitions for resistance exercises instead of three would be worthwhile in determining whether this could also bring significant improvements in our target population.

Based on current findings, twice‐a‐week yoga or tai chi sessions can be considered for at least 8 to 12 weeks for more comprehensive benefits. However, we do not know if employing protocols that include combined aerobic and resistance exercises with yoga and/or tai chi is feasible for survivors of breast cancer experiencing AIIA.

There are some limitations that were recognized in this review. Although the HOPE study used adjusted pain scores in participants taking oral medication for pain/stiffness, other studies did not specifically document or mention participant over‐the‐counter or prescription medication use for pain. Therefore, the amount of pain improvement associated with the exercise intervention versus pain medication is unable to be determined. Additionally, it may be unclear whether improvements in other AI‐associated symptoms and QoL were direct effects of the intervention itself, increased socialization through the study, or the awareness of being part of a study.

Future studies should use RCTs with larger sample sizes, focus on direct head‐to‐head comparison of different exercise modalities alone as well as in combination, and define the optimal components of the exercise prescription for use with these patients. At the same time, research efforts should seek out the reasons underlying the suboptimal adherence of these patients to the implemented exercise protocols, thus providing possible solutions to this problem.

## DISCLOSURES

There are no competing interests, funding, grants, or equipment provided for this project from any source, no financial benefits to the author, and no history of prior presentation of this case in any form.
